# Connectivity related to major brain functions in Alzheimer disease progression: microstructural properties of the cingulum bundle and its subdivision using diffusion-weighted MRI

**DOI:** 10.1186/s41747-025-00570-5

**Published:** 2025-03-19

**Authors:** Mattia Ricchi, Guido Campani, Anastasiia Nagmutdinova, Villiam Bortolotti, Danilo Greco, Carlo Golini, James Grist, Leonardo Brizi, Claudia Testa

**Affiliations:** 1 Department of Computer Science, University of Pisa, Largo Bruno Pontecorvo 3, 56127 Pisa, Italy; 2 INFN, Division of Bologna, Bologna, Italy; 3 European Institue of Oncology (IEO), Via Adamello 16, 20139 Milano, Italy; 4 Department of Civil, Chemical, Environmental, and Materials Engineering, University of Bologna, via Umberto Terracini 28, 40131 Bologna, Italy; 5 Department of Management, Economics and Industrial Engineering, Politecnico di Milano, Via Lambruschini 4/b, 20156 Milano, Italy; 6 Department of Informatics, Bioengineering, Robotics and Systems Engineering, Università Degli Studi di Genova, via Dodecaneso 35, 16146 Genova, Italy; 7 Department of Physics and Astronomy, University of Bologna, viale Berti Pichat 6/2, 40126 Bologna, Italy; 8 Department of Physiology, Anatomy and Genetics, University of Oxford, Sherrington Building Parks Road, OX13PT Oxford, England

**Keywords:** Alzheimer disease, Brain, Cognitive dysfunction, Diffusion magnetic resonance imaging, White matter

## Abstract

**Background:**

The cingulum bundle is a brain white matter fasciculus associated with the cingulate gyrus. It connects areas from the temporal to the frontal lobe. It is composed of fibers with different terminations, lengths, and structural properties, related to specific brain functions. We aimed to automatically reconstruct this fasciculus in patients with Alzheimer disease (AD) and mild cognitive impairment (MCI) and to assess whether trajectories have different microstructural properties in relation to dementia progression.

**Methods:**

Multi-shell high angular resolution diffusion imaging−HARDI image datasets from the "Alzheimer's Disease Neuroimaging Initiative"−ADNI repository of 10 AD, 18 MCI, and 21 cognitive normal (CN) subjects were used to reconstruct three subdivisions of the cingulum bundle, using a probabilistic approach, combined with measurements of diffusion tensor and neurite orientation dispersion and density imaging metrics in each subdivision.

**Results:**

The subdivisions exhibit different pathways, terminations, and structural characteristics. We found differences in almost all the diffusivity metrics among the subdivisions (*p* < 0.001 for all the metrics) and between AD *versus* CN and MCI *versus* CN subjects for mean diffusivity (*p* = 0.007–0.038), radial diffusivity (*p* = 0.008–0.049) and neurite dispersion index (*p* = 0.005–0.049).

**Conclusion:**

Results from tractography analysis of the subdivisions of the cingulum bundle showed an association in the role of groups of fibers with their functions and the variance of their properties in relation to dementia progression.

**Relevance statement:**

The cingulum bundle is a complex tract with several pathways and terminations related to many cognitive functions. A probabilistic automatic approach is proposed to reconstruct its subdivisions, showing different microstructural properties and variations. A larger sample of patients is needed to confirm results and elucidate the role of diffusion parameters in characterizing alterations in brain function and progression to dementia.

**Key Points:**

- The microstructure of the cingulum bundle is related to brain cognitive functions.
- A probabilistic automatic approach is proposed to reconstruct the subdivisions of the cingulum bundle by diffusion-weighted images.
- The subdivisions showed different microstructural properties and variations in relation to the progression of dementia.

**Graphical Abstract:**

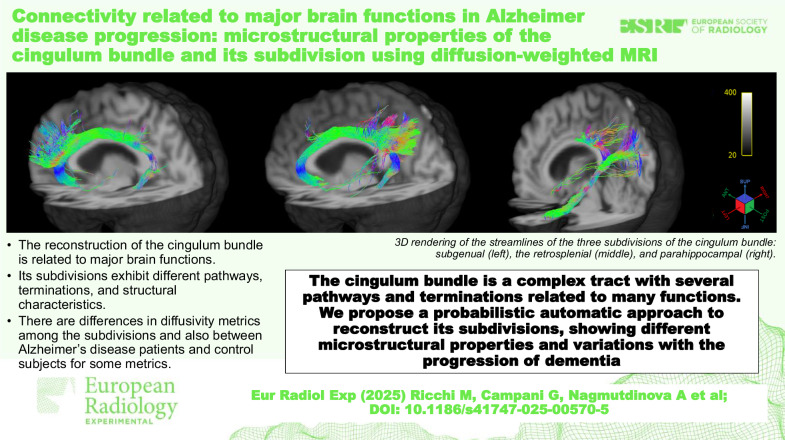

## Background

Diffusion-weighted magnetic resonance imaging (MRI) tractography is a noninvasive method used to identify anatomical connections in the brain by characterizing the water motion within the tissue. This technique is very sensitive to the presence and the orientation of fibers in the brain. If the orientation is estimated, the connections within the entire brain or between specific regions, *a priori* supposed to be connected, can be reconstructed. Several methods for the reconstruction of tracts have been developed, classified as deterministic or probabilistic [1].

Deterministic tractography algorithms assume a unique fiber orientation in each voxel and provide a single pathway emanating from each seed point; however, this approach is limited by the errors and uncertainty related to the image estimate, which may incorrectly identify the principal direction [2]. To overcome this limitation, probabilistic tractography was introduced, where a large distribution of possible trajectories is generated from each seed [2]. Although computationally more costly, it takes into account the fact that within the unit of spatial resolution (the voxel), more than one axon is present, not necessarily running parallel. Moreover, probabilistic tractography considers the possible inaccuracy of the model used for representing the diffusion signal and the error that affects the experimental measures [3].

Whole brain tractography can be used to reconstruct fiber bundles through seeding from all white matter placed throughout the brain [4]. Nevertheless, if a specific connection (a single tract) has to be reconstructed because of an a priori hypothesis, the reconstruction procedure can be performed with higher accuracy by starting from a seed point that defines a specific region of interest (ROI). Typically, these ROIs are defined either by the user or can be defined by an atlas to reduce operator dependence [5].

Here, we consider the probabilistic tractography of the cingulum bundle (CB), a highly complex tract whose structure and functions are not yet fully identified. Initial studies were conducted on rats and nonhuman primates using axonal tracers [6, 7]. Located in the medial zone of the brain, CB forms an arc connecting the parahippocampal cortex to the frontal lobe, passing through the cingulate cortex [8–10]. It is composed of short fibers within the cingulate cortex, medium-length fibers efferent and afferent to the cingulate cortex, and long fibers spanning the entire arc, each group connecting different areas. Although they all belong to the CB, each is associated with a diversity of functions [11], including emotional, reward, and motivational processes, pain processes, motor functions, conflict processes, and memory [11–13]. Tractography in humans rarely describes the complexity of CB. In the literature, it is often depicted as a single entity, uninterrupted from the temporal to the frontal lobe [14]. Correlational studies between the bundle and its associated functions are conducted by dividing the tract into different segments [15]. This approach does not allow the isolation of individual groups of fibers with common terminations, which can explain which parts of the CB are privileged for specific functions.

Recently, some attempts have been made to reconstruct different subdivisions [16], in one case having the reference standard of human brain dissections and using fiber tracking based on a high directional sampling of diffusion imaging space to achieve increased resolution of underlying white matter geometry [17]. Each subdivision is composed of fibers sharing common terminations. Moreover, the various subdivisions have different paths, but their surfaces overlap in some regions of the CB. Tractography studies have observed significant differences in microstructural properties among the various fiber populations of the different subdivisions. These differences could be attributed to the distinct functions that these subdivisions need to optimize [18, 19].

To capture these differences, a probabilistic tractography approach is better than a deterministic one since the latter has many limitations such as the inability to resolve kissing/crossing fibers. For a highly resolved reconstruction of tracts, a “high angular resolution diffusion imaging” (HARDI) acquisition protocol [20] allows the use of innovative high-order crossing fiber models, such as constrained spherical deconvolution [21] and multi-shell multi-tissue constrained spherical deconvolution [22]. Recent literature has proposed a probabilistic constrained-spherical deconvolution tractography to provide a white matter atlas of tracts, including the CB reconstruction into two subdivisions [23]. Multi-shell HARDI acquisition protocols allow the calculation of additional quantitative metrics to the standard diffusion tensor imaging (DTI) model, quantifying the deviation from the Gaussian behavior assumed in the DTI model. The “neurite orientation dispersion and density imaging” (NODDI) model proposed by Zhang et al [24] is a clinically feasible technique to estimate *in vivo* neurite orientation dispersion and density imaging evaluation. The technique combines a three-compartment tissue model with at least two-shell HARDI protocol, which calculates neurite density and orientation dispersion that can resolve two factors contributing to fractional anisotropy (FA).

The role of the CB can be of particular interest in neurodegenerative diseases whose symptoms are related to a deficit of memory, visuospatial abilities and attention, and executive functions. Among them, mild forms of dementia, like mild cognitive impairment (MCI) that can progress into Alzheimer disease (AD), present behavioral disturbances, which are considered due to dysconnectivity of specific white matter tracts [25, 26]. There is a concerted effort to find biomarkers that can predict the progression from MCI toward AD as they could be targets for possible therapies. The CB is potentially involved in the process of white matter disconnections associated with behavioral impairment in dementia. Few studies have specifically explored dysconnectivity [27–29] with DTI data from local hospitals or free databases, with the number of diffusion gradients directions from 12 to 41 using an ROI- or voxel-based approach and without attempting a specific CB reconstruction. Microscopic white matter changes with aging, and AD can be probed using advanced diffusion MRI methods [30, 31], in particular DTI or NODDI metrics.

The "Alzheimer's Disease Neuroimaging Initiative" (ADNI) repository (http://adni.loni.usc.edu/) has a small group of data in the ADNI3 repository, acquired with a multi-shell HARDI protocol in MCI, AD, and cognitively normal (CN) subjects. This is the only dataset known in literature with these technically and clinically advanced characteristics.

Our primary aim was to define an automated protocol that identifies three subdivisions of the CB and to verify the presence of fiber populations with different microstructural properties using a probabilistic tractography MRI technique. In the current literature, no study has analyzed the CB by determining its anatomical subdivisions using a multi-shell HARDI protocol, allowing the modeling of three tissue compartments (gray matter, white matter, and cerebrospinal fluid) with different diffusion characteristics to better reconstruct tracts using only the white matter compartment data. A second aim was to assess whether the CB in its subdivisions, once automatically reconstructed, has different microstructural properties in CN subjects, MCI, and AD patients. Overall, this work aimed to give a robust instrument to evaluate brain connectivity involving the CB, which can be used for the evaluation of microstructural changes in the progression of dementia.

## Methods

### Subjects and MRI protocol

The inclusion criterion was a multi-shell HARDI acquisition protocol for DTI with at least 60 gradient directions for the maximum *b*-value. We included 49 participants: 5 middle-aged CN subjects, aged 54.6 ± 2.6 years (mean ± standard deviation), 2 males and 3 females; 16 older CN subjects, aged 74.4 y ± 8.4 years, 6 males and 10 females, 18 MCI patients, aged 75.8 ± 5.4 years, 7 males and 11 females; and 10 AD, aged 76.1 ± 7.9 years, 4 males and 6 females from the ADNI database. Subjects completed the Mini-Mental State Exam (MMSE) and Montreal cognitive assessment (MoCA) [32, 33]. The characteristics of CN, MCI, and AD subjects are shown in detail in Table 1. Sagittal 3D T1-weighted anatomical images were acquired. Details on technical parameters are provided in Supplementary materials, Methods).

**Table 1:** Subjects and cognitive scores characteristics

	Number (females)	Age, years (mean ± standard deviation)	MMSE^a^	MoCA^b^
Cognitively normal (CN)	16 (10)	74.4 ± 8.4	28.4 ± 1.4	25.2 ± 2.0
Mild cognitive impairment (MCI)	18 (11)	75.8 ± 5.4	27.2 ± 1.9	21.1 ± 4.2
Alzheimer disease (AD)	10 (6)	76.1 ± 7.9	23.7 ± 4.8	16.0 ± 3.8
*χ*^2^ (*p*-value)	0.992			
ANOVA (*p*-value)		0.801	0.001	< 0.001
Tukey HSD (*post hoc* test)				
AD < CN (adjusted *p*-value)			< 0.001	< 0.001
AD < MCI (adjusted *p*-value)			0.015	0.009
MCI < CN (adjusted *p*-value)			0.453	0.006

*MMSE* Mini-Mental State Exam, *MoCA* Montreal cognitive assessment

^a^ MMSE was available for 9 out of 10 AD, for 16 out of 19 MCI

^b^ MoCA was available for 7 out of 10 AD, for 15 out of 19 MCI

### DTI processing and image registration

DTI data were preprocessed using a self-developed automated workflow based on software packages freely available as part of the Oxford Functional Magnetic Resonance Imaging Software Library (FSL) [34] version 6, MRtrix3 version 3.0.2 [35, 36] and using a recent tool Synb0-DISCO [37, 38] (see Supplementary materials, Methods).

Microstructural properties of the brain tissue were characterized by estimating voxel FA, mean diffusivity (MD), and radial diffusivity (RD) using FSL. The implementation of the Bingham-NODDI model was performed by using the Diffusion Microstructure Imaging in Python (Dmipy) package [39] to calculate the NODDI metrics: tissue volume fraction, *β*-fraction, neurite dispersion index (NDI), and orientation dispersion index (ODI) (see Supplementary materials, Methods).

The image alignments to the diffusion space and to the T1 space have been performed by FSL registration tools both for linear and nonlinear registration (see Supplementary materials, Methods).

### CB tract reconstruction: methods

Tractography was performed by *tckgen* which generates streamlines using a first-order integration over fiber orientation distributions (*iFOD2*) approach [40].

Firstly, the CB reconstruction was performed in the middle-aged CN group to identify the ROIs for the automatic reconstruction, to understand the feasibility of the method, and to explore the robustness of the approach. Then, the methodology was applied to the three groups of subjects. Streamline integration utilized the *iFOD2* algorithm starting from two-dimensional ROIs. “Include” and “exclude” ROIs were used. For each hemisphere, we reconstructed three different subdivisions of the CB as Jones et al described in their work [16]. We named the three tracts as follows: subgenual cingulum (SGC); retrosplenial cingulum (RSC); and parahippocampal cingulum (PHC).

Details about the reconstruction of the subdivisions in the middle-aged CN group are reported in Supplementary material, Methods—Supplemental Fig. S1, Table S1, Fig S2.

### Cingulum bundle tract reconstruction: application to subjects’ groups

Once the ROIs and the masks were defined in the MNI space (see Supplemetal material, Methods), the methods for the CB reconstructions were applied to the subdivisions, across subjects. For new subjects, the ROIs and the masks were registered from the MNI space in their own diffusion space, and streamlines were constructed for each of the three subdivisions of the CB.

The alignment of the ROIs in the diffusion space was checked and in no case we had to re-define them. The resulting streamlines were then trimmed according to the boundaries defined by the mask, which was binarized and multiplied for the corresponding reconstructed tract, following a method already validated [5, 41].

### Statistical analysis

An ANOVA was used to control groups’ age, with *p* = 0.050 as a cutoff for significance. The sex distribution in the subjects’ groups was tested using a *χ*^2^ test. Tracts were reconstructed for all 49 participants. The mean values within each tract subdivision of DTI and NODDI metrics were then compared and analyzed. Firstly, the normality and homogeneity of variances of the distributions of FA, MD, RD, tissue volume fraction, *β*-fraction, NDI, and ODI values for each group and each subdivision were tested using the Shapiro-Wilks and Levene’s tests. Then, the distributions of microstructural values were compared among groups, tract subdivisions of the same type but located in different hemispheres (side), as well as the distributions of values for different tracts. This compatibility test was performed using the three-way factor ANOVA test. In these cases, the *post hoc* test, Tukey’s honestly significant difference (HSD) test, was employed. *Post hoc* tests control the family-wise error rate by the Benjamini–Hochberg step-up procedure [42]. A Pearson correlation analysis was performed for the mean values within each tract subdivision of all the diffusion metrics and MoCA and MMSE scores. Python libraries were used for statistical analysis (Python 3.11.9).

## Results

The code of the pipeline, the MNI ROIs and masks have been shared in a Github repository (https://github.com/MattRicchi/Cingulum_Bundle_tractography.git).

The three groups—CN, MCI, and AD—did not differ significantly in age (ANOVA, *p* = 0.801) or gender (*χ*^2^, *p* = 0.992) but did differ in MMSE (ANOVA: *p* = 0.001) and in MoCA (ANOVA, *p* < 0.001). The *post hoc* analysis showed that there is a significant difference between AD and CN and between AD and MCI for the MMSE score as well as between all the pairs of subjects’ groups for the MoCa score. Demographic results are shown in Table 1.

The use of Synb0-DISCO was successfully applied to all the diffusion-weighted images and results were evaluated as optimal after performing the image difference between pre- and post-correction, and visually assessing that the regions affected by distortions were those corrected by the algorithm (Supplemental Fig. S3).

**Fig. 1:**
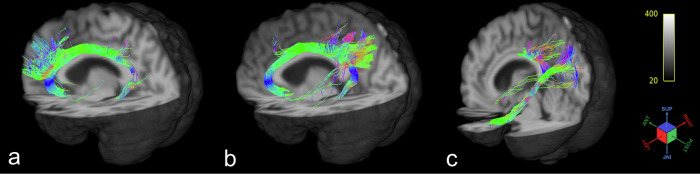
Three-dimensional rendering of the streamlines of the three subdivisions of the cingulum bundle for one middle-aged cognitive normal case (female, 53-y.o.): **a** the subgenual subdivision without the application of the mask; **b** the retrosplenial subdivision without the application of the mask; **c** the parahippocampal subdivision without the application of the mask (streamlines that may be false-positive findings are visible as isolated fibers)

Tracts were reconstructed for all the subjects except for one hemispherical SGC subdivision in one patient of the AD group. This failure was thought to be due to highly enlarged ventricles and specifically with a substantial asymmetry in the compartments. An example of the reconstruction of the three subdivisions for one middle-aged CN case is shown in Fig. 1.

### Tractography

**Fig. 2:**
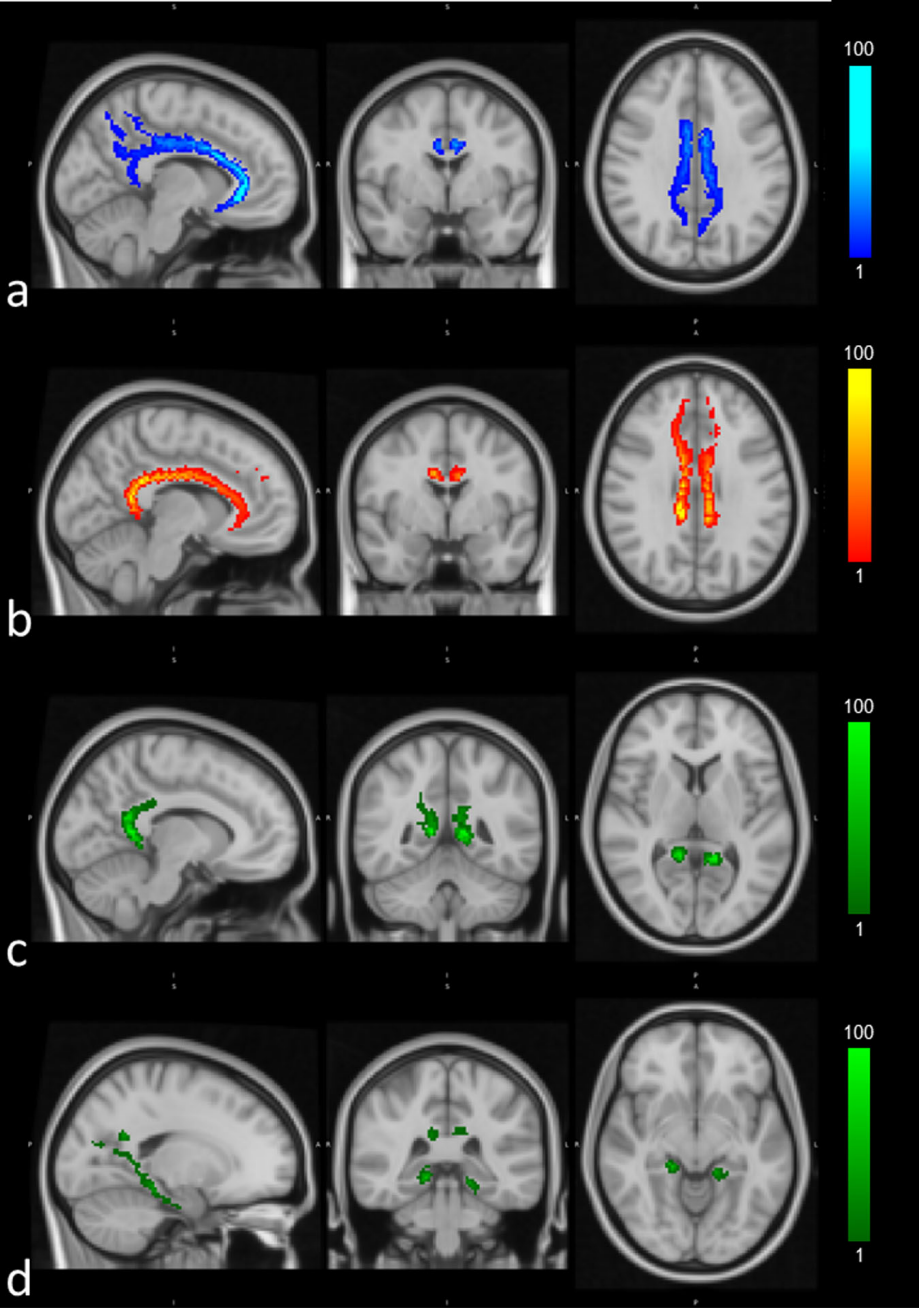
Orthogonal view of the three subdivisions of the CB reconstructed for one middle-aged cognitive normal case (female, 53-y.o.) aligned to the Montreal Neurological Institute atlas. The signal (fiber *orientation distribution function*) is normalized between 0 and 100. **a** The SGC subdivision shows this portion of the CB going from the orbitofrontal cortex to the rostral cingulate cortex, extending above the corpus callosum. The terminations of the bundle are around the posterior cingulate cortex and near the ventral precuneus area. **b** The RSC originates from the rostral cingulate cortex, the level of the most anterior point of the corpus callosum extending above the corpus callosum and terminating in the isthmus of the cingulate gyrus. **c**, **d** The PHC subdivision connects the hippocampal and parahippocampal areas (**d**) up to the region of the cortex between the precuneus and the isthmus of the cingulate gyrus (**c**). CB, Cingulum bundle; PHC, Parahippocampal cingulum; RSC, Retrosplenial cingulum; SGC, Subgenual cingulum

The SGC subdivision starts anteriorly from the lateral orbitofrontal cortex, medial orbitofrontal cortex, and rostral cingulate cortex. It extends superiorly to the corpus callosum, slightly shifting towards the sagittal plane. The terminations of the bundle are around the posterior cingulate cortex and near the ventral precuneus area. The RSC also originates from the rostral cingulate cortex, but in a more superior position, at the level of the most anterior point of the corpus callosum. The bundle then extends above the corpus callosum and terminates in the isthmus of the cingulate gyrus. The PHC subdivision connects the hippocampal and parahippocampal areas up to the region of the cortex between the precuneus and the isthmus of the cingulate gyrus (Fig. 2).

### Microstructural values

**Fig. 3:**
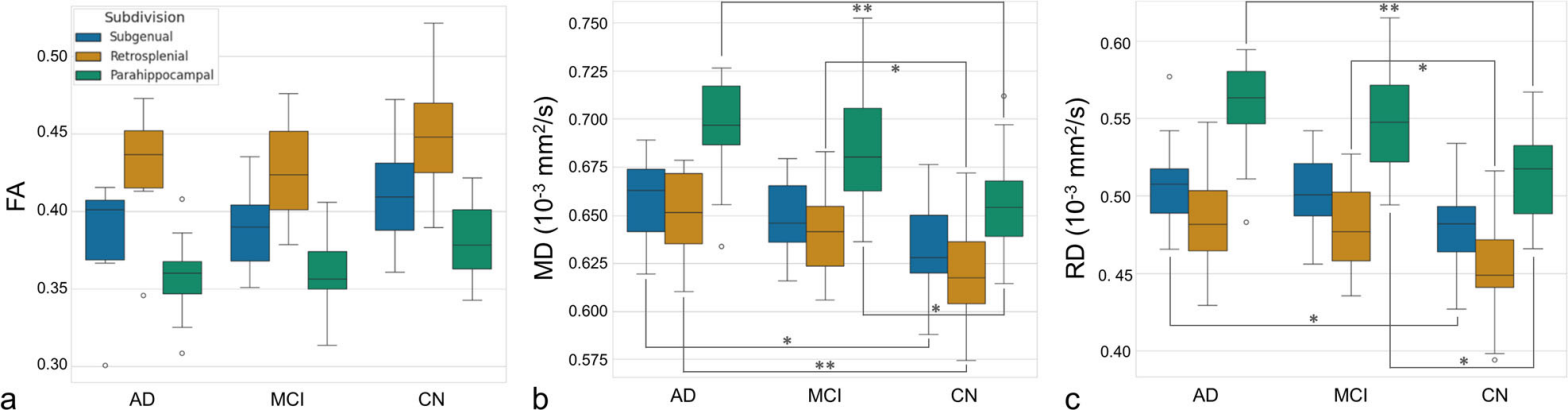
Box-plots of the distribution FA (**a**), MD (**b**), and RD (**c**) values for the three subdivisions of the cingulum bundle and the three groups of subjects. * indicates significant differences with a *p*-value < 0.05; ** indicates significant differences with a *p*-value < 0.01. AD, Alzheimer disease; CN, Cognitive normal; FA, Fractional anisotropy; MCI, Mild cognitive impairment; MD, Mean diffusivity; RD, Radial diffusivity

**Fig. 4:**
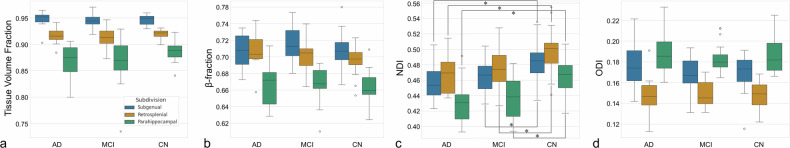
Box-plots of the distribution total volume fraction (**a**), *β*-fraction (**b**), NDI (**c**), and ODI (**d**) values for the three subdivisions of the cingulum bundle and the three groups of subjects. * indicates significant differences with a *p*-value < 0.05; ** indicates significant differences with a *p*-value < 0.01. AD, Alzheimer disease; CN, Cognitive normal; MCI, Mild cognitive impairment; NDI, Neurite density index; ODI, Orientation dispersion index

Figures 3 and 4 show the boxplots of the distributions of FA, MD, and RD values and for the NODDI metrics respectively for the three subdivisions and the three groups of subjects. The normality and homogeneity of variances tests suggested that the data for each group of patients did not significantly deviate from a normal distribution (*p* = 0.116 for AD, *p* = 0.699 for MCI, and *p* = 0.246 for CN).

A three-way ANOVA test was performed for FA, MD, and RD (Table 2). A significant effect was found on all the DTI metrics for the subdivisions and groups: FA subdivision effect, *F* = 90.58, *p* < 0.001; FA group effect, *F* = 11.83; *p* < 0.001; MD subdivision effect, *F* = 61.34, *p* < 0.001; MD group effect, *F* = 31.25, *p* < 0.001; RD subdivision effect, *F* = 93.09, *p* < 0.001; and RD group effect, *F* = 25.96, *p* < 0.001.

**Table 2:** Three-way ANOVA results for the three metrics: fractional anisotropy, mean diffusivity, and radial diffusivity

Fractional anisotropy			Mean diffusivity			Radial diffusivity		
Effect	*F*	*p*-value	Effect	*F*	*p*-value	Effect	*F*	*p*-value
Subdivision (SGC, RSC, PHC)	90.58	< **0.001**	Subdivision (SGC, RSC, PHC)	61.34	< **0.001**	Subdivision (SGC, RSC, PHC)	93.09	< **0.001**
Side (left, right)	0.30	0.579	Side (left, right)	0.96	0.329	Side (left, right)	0.03	0.864
Group (AD, MCI, CN)	11.83	< **0.001**	Group (AD, MCI, CN)	31.25	< **0.001**	Group (AD, MCI, CN)	25.96	< **0.001**
Subdivision : Group	0.17	0.953	Subdivision : Group	0.42	0.792	Subdivision : Group	0.26	0.901
Subdivision : Side	1.20	0.301	Subdivision : Side	1.70	0.184	Subdivision : Side	1.49	0.226
Side : Group	0.66	0.513	Side : Group	0.24	0.789	Side : Group	0.38	0.685
Subdivision : Tract : Side	0.05	0.995	Subdivision : Tract : Side	0.01	0.999	Subdivision : Tract : Side	0.02	0.999

*AD* Alzheimer disease, *CN* Cognitively normal, *MCI* Mild cognitive impairment, *PHC* Parahippocampal cingulum, *RSC* Retrosplenial cingulum, *SGC* Subgenual cingulum

In bold the significant adjusted *p*-values (< 0.05)

In addition, the same test was performed for tissue volume fraction, *β*-fraction, NDI, and ODI (Table S2). A significant effect for subdivision was found on all the NODDI metrics. In addition, a significant group effect was found for tissue volume fraction and NDI: tissue volume fraction subdivision effect, *F* = 187.01, *p* < 0.001; tissue volume fraction group effect, *F* = 3.72, *p* = 0.026; *β*-fraction subdivision effect, *F* = 75.80, *p* < 0.001; NDI subdivision effect, *F* = 39.74, *p* < 0.001; NDI group effect, F = 25.86, *p* < 0.001; and ODI subdivision effect, *F* = 74.15, *p* < 0.001. A side effect (and a causal interaction effect between side and subdivisions) was found for *β*-fraction (*F* = 8.46, *p* < 0.004). Being the only diffusion metric sensitive to the side effect, it was not taken into account in this study, deserving further investigation. For this reason, microstructural parameters were then averaged between left and right.

*Post hoc* analyses were performed independently for the group effect and for the subdivision effect given the results of ANOVA. The Tukey’s HSD test was performed applying the Benjamini–Hochberg correction. Table 3 and Supplemental Table S3 show the results for group *post hoc* comparison for the DTI and NODDI metrics, respectively. Table 3 and Supplemental Table S3 show that the lowest effect size relative to the AD *versus* MCI is FA in the RSC subdivision (effect size = 0.11).

**Table 3:** *Post hoc* analyses for group differences performed by the Tukey’s HSD test applying the Benjamini–Hochberg correction for the adjusted *p*-values

Fractional anisotropy				Mean diffusivity				Radial diffusivity			
Group	Subdivision	Adjusted *p*-value	Effect size	Group	Subdivision	Adjusted *p*-value	Effect size	Group	Subdivision	Adjusted *p*-value	Effect size
AD < CN	SGC	0.169	0.71	AD > CN	SGC	**0.019**	1.08	AD > CN	SGC	**0.049**	0.94
AD < MCI	SGC	0.926	0.15	AD > MCI	SGC	0.622	0.40	AD > MCI	SGC	0.833	0.24
MCI < CN	SGC	0.185	0.65	MCI > CN	SGC	0.062	0.83	MCI > CN	SGC	0.070	0.82
AD < CN	RSC	0.347	0.51	AD > CN	RSC	**0.009**	1.16	AD > CN	RSC	0.052	0.89
AD < MCI	RSC	0.963	0.11	AD > MCI	RSC	0.603	0.41	AD > MCI	RSC	0.941	0.14
MCI < CN	RSC	0.138	0.68	MCI > CN	RSC	**0.038**	0.89	MCI > CN	RSC	**0.047**	0.86
AD < CN	PHC	0.077	0.88	AD > CN	PHC	**0.007**	1.35	AD > CN	PHC	**0.008**	1.33
AD < MCI	PHC	0.880	0.19	AD > MCI	PHC	0.691	0.31	AD > MCI	PHC	0.737	0.28
MCI < CN	PHC	0.109	0.75	MCI > CN	PHC	**0.018**	0.99	MCI > CN	PHC	**0.018**	1.01

*AD* Alzheimer disease, *CN* Cognitively normal, *MCI* Mild cognitive impairment, *PHC* Parahippocampal cingulum, *RSC* Retrosplenial cingulum, *SGC* Subgenual cingulum

In bold the significant adjusted *p*-values (< 0.05). Effect size computed as “*Cohen’s d*” (small = 0.2; medium = 0.5; large: 0.8

The *post hoc* analysis showed that FA and tissue volume fraction were not significantly different for the group effect. MD, RD, and NDI showed a significant effect for the comparison AD *versus* CN in almost all the subdivisions and for the comparison MCI *versus* CN in the RSC subdivision.

The same post-hoc analysis was performed considering the subdivisions for the DTI and NODDI metrics respectively (Table 4 and Supplemental Table S4). Almost all the diffusion metrics were significant for the subdivision effect. Only for the comparisons of RSC *versus* SGC (in each group), diffusion metrics result in less sensitive.

**Table 4:** *Post hoc* analyses for subdivision differences performed by the Tukey’s HSD test applying the Benjamini–Hochberg correction for the adjusted *p*-value

Fractional anisotropy			Mean diffusivity			Radial diffusivity		
Subdivision	Group	Adjusted *p*-value	Subdivision	Group	Adjusted *p*-value	Subdivision	Group	Adjusted *p*-value
RSC > PHC	CN	**<** **0.001**	RSC < PHC	CN	**<** **0.001**	RSC < PHC	CN	**<** **0.001**
SGC > PHC	CN	**0.035**	SGC < PHC	CN	**0.019**	SGC < PHC	CN	**0.008**
RSC > SGC	CN	**0.003**	RSC < SGC	CN	0.418	RSC < SGC	CN	**0.044**
RSC > PHC	AD	**<** **0.001**	RSC < PHC	AD	**0.003**	RSC < PHC	AD	**<** **0.001**
SGC > PHC	AD	0.202	SGC < PHC	AD	**0.019**	SGC < PHC	AD	**0.019**
RSC > PHC	AD	**0.021**	RSC < SGC	AD	0.781	RSC < SGC	AD	0.238
RSC > PHC	MCI	**<** **0.001**	RSC < PHC	MCI	**<** **0.001**	RSC < PHC	MCI	**<** **0.001**
SGC > PHC	MCI	**0.009**	SGC < PHC	MCI	**<** **0.001**	SGC < PHC	MCI	**<** **0.001**
RSC > SGC	MCI	**<** **0.001**	RSC < SGC	MCI	0.510	RSC < SGC	MCI	**0.047**

*AD* Alzheimer disease, *CN* Cognitively normal, *MCI* Mild cognitive impairment, *PHC* Parahippocampal cingulum, *RSC* Retrosplenial cingulum, *SGC* Subgenual cingulum

In bold the significant adjusted *p*-values (< 0.05)

Correlation analysis between diffusion metrics and MoCA scores for each tract subdivision showed no statistically significant results after the correction for multiple comparisons. Nevertheless, the MD and NDI values for the PHC subdivision correlated with MoCA score with an uncorrected *p* = 0.030 and *p* = 0.049, respectively; for the same subdivision RD had *p* = 0.041. The RSC subdivision MD was significant with an uncorrected *p*-value of 0.047.

Correlation analysis between diffusion metrics and MMSE scores was explored even if the Tukey HSD *post hoc* test did not reach significant results for the MCI *versus* CN comparison. Results were not statistically significant after the correction for multiple comparisons. Nevertheless, the MD and NDI values for the PHC subdivision correlated with the MMSE score with an uncorrected *p* = 0.027 and *p* = 0.034, respectively.

## Discussion

We have proposed an automatic protocol to explore the connectivity of the CB, retracing the steps reported by Jones et al [16] suitable to reconstruct the three subdivisions: SGC, RSC, and PHC. Differently from these authors, we used a probabilistic approach ensuring a more robust, comprehensive, and reliable representation of the fibers. Successive research both in humans and macaques [17, 43] has compared CB reconstruction with brain dissection, which, even if not wholly safe from false findings, is the reference standard for tractography. Hailbronner and Haber [43] grouped the fibers of CB into four zones based on their specific connections: subgenual, rostral dorsal, caudal dorsal, and temporal.

The presence of fibers that do not originate or terminate in the cingulate cortex, has reshaped the understanding of the CB. It is not seen solely as fibers associated with the cingulate gyrus, but it is a multilayered network of connection with short, medium, and long-length fibers that share different patterns of connection. Wu et al [17] performed a deterministic tractography compared with brain dissections. According to this work, the CB should be divided into five components: CB-I to CB-V briefly are analogous to our SGC, RSC, and PHC; CB-II and CB-III corresponding to our RSC; CB-IV, a minor subcomponent from the superior parietal lobule and precuneus to the supplementary and premotor areas in the frontal region of the brain has not an exact correspondence to our subdivisions. Our approach was not suitable for reproducing these five subdivisions even though the three subdivisions—SGC, RSC, and PHC—can be associated with compositions of the five subdivisions obtained through dissection reported by Wu et al [17]. Nevertheless, our approach relies on a multishell HARDI protocol, which, combined with a multi-tissue probabilistic approach, has been demonstrated to be more robust in the reconstruction of kissing and crossing fibers [2, 40, 44]. Moreover, we have applied a multi-compartment model to calculate diffusion microstructural properties measuring the NODDI metrics.

Our workflow allowed us to reconstruct the CB subdivisions for all the subjects with the exception of one subdivision out of 294 (3 subdivisions, both for the left and right hemispheres and for 49 subjects) due to high asymmetrical enlargement of ventricles. We selected a homogeneous set of data for which the CB reconstruction is clinically relevant and with acquisition parameters of the DTI sequence corresponding to a multi-shell HARDI protocol. The limit of this dataset due to the lack of specific sequences for distortion correction has been successfully overcome by using the Synb0-DISCO tool [38] (Supplemental Fig. S3). This tool opens the opportunity to use many other public data repositories for brain connectivity research, even if the quality of diffusion-weighed images is not optimal for tractography reconstruction.

The workflow can be easily reproduced using the set of ROIs explicitly drawn and registered in the MNI space to make the procedure automatic (Supplemental Fig. S1 and Supplemental Table S1). A definition of a mask (Supplemental Fig. S2) to trim the streamlines for each subdivision has been demonstrated to work efficiently to reduce the few false positives (Fig. 1).

The spatial disposition of the subdivisions is different for each of them. The subgenual subdivision is the most medial and frontal one; it starts from the lateral-medial orbital cortex and continues above the corpus callosum to the caudal limit of the posterior cingulate cortex and near the ventral precuneus area (Fig. 1a). It can be associated with CB-I described by Wu et al [17]. Due to its position, as suggested by Wu et al, this bundle could be linked to emotional responses to painful stimuli [45], cognitive functions, and verbal memory performance [46]. Its termination inside the precuneus may have a fundamental role in the default mode network, according to Skandalakis et al [47]. Finally, it may be related to the executive function: Bubb et al [15] reviewed the literature reporting correlations between FA and MD and working memory, attention, and executive functions and found that significant results exist only for the anterior and posterior portion of the CB in healthy elderly subjects [48], so precisely the terminations covered by this subdivision.

The RSC subdivision, which wraps around the corpus callosum in a semicircle, is positioned more laterally than the SGC subdivision and can be associated with CB-II and CB-III [15] (Fig. 1b). Due to its connections, this tract could be associated with the response conflict [49] and the motor function [11]. Referring to the study of Fox et al [50], this tract may play a significant role in the performance of demanding cognitive tasks or the integration pathways [50, 51].

The PHC subdivision starts from the precuneus area and the isthmus cingulate cortex, terminating in the temporal lobe near the parahippocampal cortex. It is slightly more lateral than the RSC subdivision (Fig. 1c). This subdivision can be associated with CB-V described by Wu et al [17]. The CB-V is believed to function as a regulating route that facilitates various cognitive abilities, encompassing the transmission of visuospatial, facial, and memory cues from the medial temporal region to the precuneus [47].

In this work, we have shown that the two hemispheres had no statistically significant difference in tensor and NODDI metrics with the exception of *β*-fraction for all the subdivisions, while for each of the diffusion metrics, there are differences between subdivisions and groups of patients (Table 2 and Supplemental Table S2). The significance of the *β*-fraction has been neglected in the analysis, deserving a deeper analysis in a larger cohort of healthy subjects.

The three subdivisions have shown highly significant differences for almost all the pair comparisons of FA, RD, tissue volume fraction, and ODI and in the two pair comparisons involving the PHC subdivision of the MD (Table 4 and Supplemental Table S4). FA and tissue volume fraction showed the lowest value for the PHC subdivision, possibly for the high curvature of the tract and for the closeness to the fourth ventricles (Fig. 2c and Fig. 4a, respectively). The RSC subdivision showed the highest FA values, possibly due to the high orientation/low spread of this portion of the CB, and a lower tissue volume fraction with respect to the SGC subdivision, possibly because the RSC is closer to the lateral ventricles. These trends are inversely reflected in the MD and RD trends. Thus, the effort made to reconstruct the CB representing a multilayered network of short, medium, and long-length fibers that share different patterns of connection is useful to characterize the specific microstructural properties of each of them. These results are consistent with findings described by Beckmann et al [11], indicating that each subdivision of the same type identifies populations of fibers with different structural characteristics.

Concerning differences between groups (Table 3 and Supplemental Table S3), we found that FA and tissue volume fraction were not significantly sensitive to CB neurodegeneration for all the subdivisions in this data set. The adjusted *p*-value was close to the significance threshold and corresponds to the comparison of AD *versus* CN for the parahippocampal subdivision. The PHC subdivision was the most impaired in terms of MD RD and NDI for both the comparisons of AD and MCI with CN. The comparisons between AD and CN of MD and NDI values showed significant differences also for SGC and RSC.

Overall, MD, RD, and NDI were the most affected metrics reflecting possible early axonal degeneration. Moreover, the PHC subdivision is the most sensitive to degeneration due to the AD, also considering the trend of correlation between the MoCA and MMSE score and MD, RD, and NDI in this subdivision. This is in line with the fact that the PHC subdivision has its term in the parahippocampal and hippocampal gray matter regions that are the most affected by degeneration in AD patients [52].

Some limitations can be considered, essentially related to the small number of MCI and AD patients. In fact, although ADNI is the most extensive data repository for neuroimages in dementia, the type of data we have chosen is the best to apply a multi-shell multi-tissue analysis of diffusion-weighted images, but they were few. This may have conditioned the nonsignificant differences between MCI and AD for both the DTI and NODDI metrics. As shown in Table 3, if we consider at least a small effect size of 0.2, a power analysis assesses that a minimum number of 26 MCI *versus* 26 AD patients is needed to detect a statistically significant difference of FA in this comparison. This number would ensure a small effect size for almost all the other metrics comparisons.

An extensive neuropsychological battery of tests to assess a wide range of cognitive functions would be necessary for patients affected with dementia to evaluate which subdivision is primarily sensitive to one specific cognitive impairment and if this evaluation predicts the progression of dementia. Future work will be devoted to applying the presented methodology to datasets with multi-shell HARDI protocol which is already used in the clinical presurgical setting [53, 54].

In conclusion, this study contributes to aligning the CB representation with its anatomical and biological understanding. We have developed a probabilistic tractography protocol using high-resolution diffusion-weighted images for the automatic determination of the three subdivisions of the CB. They exhibited different terminations and pathways with distinct structural characteristics. The overall result plays in favor of future correlational studies in the realm of functions and analyzes specific markers for certain pathologies: the subgenual tract for executive functions and the default mode network; the retrosplenial tract for motor functions and conflict response processes as well as demanding cognitive tasks or integration pathways; the parahippocampal tract for the transmission of visuospatial, facial, and memory cues.

## Supplementary information

**Additional file 1: Fig. S1.** Three-dimensional rendering of the ROIs used to reconstruct the three subdivisions of the cingulum bundle superimposed in the Montreal Neurological Institute space represented as a surface. ROI 1 (violet) and ROI 2 (teal) were used for the reconstruction of the subgenual subdivision as seed and “include” mode, respectively; ROI 3 (red) and ROI 4 (green) were used for the reconstruction of the retrosplenial subdivision as seed and “include” mode, respectively; ROI 5 (blue), ROI 6 (pink for the left hemisphere and yellow for the right hemisphere), and ROI 4 were used to reconstruct the parahippocampal subdivision as seed, “exclude”, and “include” mode, respectively. *ROI* Region of interest. **Fig. S2.** Orthogonal view of the thresholded (at 1%) overlap of the subdivisions of the cingulum bundle for the five middle-aged cognitive normal case (average representation): subgenual in cyan-blue color map (**a**); retrosplenial in red-yellow color map (**b**); and parahippocampal in light-dark green color map (**c**). The intensity scales are percentages associated to the minimum and maximum of the subjects represented by the overlap of the tracts. **Fig. S3.** (**a**) Example of axial views of diffusion-weighted images (*b* = 0 s/mm2) before distortion correction; distortions are evident especially in the frontal part of the brain. (**b**) The same axial view after Synb0-DISCO application and distortions correction, showing the frontal part of the brain restored. (**c**) The difference between **a** and **b** showing at a first glance, regions were the Synb0-DISCO have mostly corrected the distortions. **Table S1.** Coordinates of the center of gravity of the six ROIs and the midsagittal ROI used to reconstruct the subgenual, retrosplenial, parahippocampal subdivision of the cingulum bundle are reported in standard Montreal Neurological Institute space for both hemispheres. **Table S2.** Three-way ANOVA results for four NODDI metrics: tissue volume fraction, β-fraction, neurite dispersion index, and orientation dispersion index. **Table S3.** Post hoc analyses for group differences performed by the Tukey’s HSD test applying the Benjamini–Hochberg correction for the adjusted *p*-values. **Table S4.** Post hoc analyses for subdivision differences performed by the Tukey’s HSD test applying the Benjamini–Hochberg correction for the adjusted *p*-values.

## Data Availability

Data used were obtained from the ADNI database (adni.loni.usc.edu). ADNI was launched in 2003 as a public-private partnership, led by Principal Investigator Michael W. Weiner, MD. The primary goal was to test whether serial MRI, positron emission tomography, other biological markers, and clinical and neuropsychological assessment can be combined to measure the progression of MCI and early Alzheimer’s disease. For more information, see www.adni-info.org. Publicly available datasets were analyzed in this study. This data can be found here: http://adni.loni.usc.edu/. Data used in the preparation of this article were obtained from Alzheimer’s Disease. Neuroimaging Initiative (ADNI) database (adni.loni.usc.edu). As such, the investigators within the ADNI contributed to the design and implementation of ADNI and/or provided data but did not participate in the analysis or writing of this report. A complete listing of ADNI investigators can be found at: http://adni.loni.usc.edu/wp-content/uploads/how_to_apply/ADNI_Acknowledgement_List.pdf.

## Abbreviations

AD: Alzheimer disease
ADNI: Alzheimer Disease Neuroimaging Initiative
CB: Cingulum bundle
CN: Cognitively normal
DTI: Diffusion tensor imaging
FA: Fractional anisotropy
FSL: Oxford Functional Magnetic Resonance Imaging Software Library
HARDI: High angular resolution diffusion imaging
HSD: Honestly significant difference
MCI: Mild cognitive impairment
MD: Mean diffusivity
MMSE: Mini-Mental State Exam
MNI: Montreal Neurological Institute
MoCA: Montreal cognitive assessment
MRI: Magnetic resonance imaging
NDI: Neurite dispersion index
NODDI: Neurite orientation dispersion and density index
ODI: Orientation dispersion index
PHC: Parahippocampal cingulum
RSC: Retrosplenial cingulum
RD: Radial diffusivity
ROI: Region of interest
SGC: Subgenual cingulum

## References

[1] Bastiani M (2012) Human cortical connectome reconstruction from diffusion weighted MRI: the effect. Neuroimage 62:1732–1749. 10.1016/j.neuroimage.2012.06.002 22699045 10.1016/j.neuroimage.2012.06.002

[2] Talozzi L, Testa C, Evangelisti S et al (2018) Along-tract analysis of the arcuate fasciculus using the Laplacian operator to evaluate different tractography methods. Magn Reson Imaging 54:183–193. 10.1016/j.mri.2018.08.013 30165094 10.1016/j.mri.2018.08.013

[3] Woolrich MW, Jbabdi S, Patenaude B et al (2009) Bayesian analysis of neuroimaging data in FSL. NeuroImage 45:S173–S186. 10.1016/j.neuroimage.2008.10.055 19059349 10.1016/j.neuroimage.2008.10.055

[4] Sporns O (2013) Structure and function of complex brain networks. Dialog Clin Neurosci 15:247–262. 10.31887/DCNS.2013.15.3/osporns 10.31887/DCNS.2013.15.3/osporns PMC3811098 24174898

[5] Fiscone C, Sighinolfi G, Manners DN et al (2024) Multiparametric MRI dataset for susceptibility-based radiomic feature extraction and analysis. Sci Data 11:575. 10.1038/s41597-024-03418-6 38834674 10.1038/s41597-024-03418-6 PMC11150520

[6] Vogt BA, Deepak NP (1987) Cingulate cortex of the rhesus monkey: II. Cortical afferents. J Comp Neurol 262:271–289. 10.1002/cne.902620208 3624555 10.1002/cne.902620208

[7] Mufson EJ, Deepak NP (1984) Some observations on the course and composition of the cingulum bundle in the rhesus monkey. J Comp Neurol 225:31–43. 10.1002/cne.902250105 6725639 10.1002/cne.902250105

[8] Catani M, Howard RJ, Pajevic S, Jones DK (2002) Virtual in vivo interactive dissection of white matter fasciculi in the human brain. Neuroimage 17:77–94. 10.1006/nimg.2002.1136 12482069 10.1006/nimg.2002.1136

[9] Catani M, Thiebaut de Schotten M (2012) Atlas of human brain connections. Oxford University Press, USA

[10] Singh M, Wong CW (2010) Independent component analysis-based multifiber streamline tractography of the human brain. Magn Reson Med 64:1676–1684. 10.1002/mrm.22509 20882674 10.1002/mrm.22509 PMC3062917

[11] Beckmann M, Johansen-Berg H, Rushworth MF (2009) Connectivity-based parcellation of human cingulate cortex and its relation to functional specialization. J Neurosci 29:1175–1190. 10.1523/JNEUROSCI.3328-08.2009 19176826 10.1523/JNEUROSCI.3328-08.2009 PMC6665147

[12] Rudebeck PH, Buckley MJ, Walton ME, Rushworth MFS (2006) A role for the macaque anterior cingulate gyrus in social valuation. Science 313:1310–1312. 10.1126/science.1128197 16946075 10.1126/science.1128197

[13] Kennerley SW, Walton ME, Behrens TE, Buckley MJ, Rushworth MF (2006) Optimal decision making and the anterior cingulate cortex. Nat Neurosci 9:940–947. 10.1038/nn1724 16783368 10.1038/nn1724

[14] Bettcher BM, Mungas D, Patel N et al (2016) Neuroanatomical substrates of executive functions: beyond prefrontal structures. Neuropsychologia 85:100–109. 10.1016/j.neuropsychologia.2016.03.001 26948072 10.1016/j.neuropsychologia.2016.03.001 PMC4853308

[15] Bubb EJ, Metzler-Baddeley C, Aggleton JP (2018) The cingulum bundle: anatomy, function, and dysfunction. Neurosci Biobehav Rev 92:104–127. 10.1016/j.neubiorev.2018.05.008 29753752 10.1016/j.neubiorev.2018.05.008 PMC6090091

[16] Jones DK, Christiansen KF, Chapman RJ, Aggleton JP (2013) Distinct subdivisions of the cingulum bundle revealed by diffusion MRI fibre tracking: implications for neuropsychological investigations. Neuropsychologia 51:67–78. 10.1016/j.neuropsychologia.2012.11.018 23178227 10.1016/j.neuropsychologia.2012.11.018 PMC3611599

[17] Wu Y, Sun D, Wang Y, Wang Y, Ou S (2016) Segmentation of the cingulum bundle in the human brain: a new perspective based on DSI tractography and fiber dissection study. Front Neuroanat 10:84. 10.3389/fnana.2016.00084 27656132 10.3389/fnana.2016.00084 PMC5013069

[18] Cannistraro PA, Makris N, Howard JD et al (2007) A diffusion tensor imaging study of white matter in obsessive–compulsive disorder. Depress Anxiety 24:440–446. 10.1002/da.20246 17096398 10.1002/da.20246

[19] Wise T, Radua J, Nortje G, Cleare AJ, Young AH, Arnone D (2016) Voxel-based meta-analytical evidence of structural disconnectivity in major depression and bipolar disorder. Biol Psychiat 79:293–302. 10.1016/j.biopsych.2015.03.004 25891219 10.1016/j.biopsych.2015.03.004

[20] Tuch DS, Reese TG, Wiegell MR, Makris N, Belliveau JW, Wedeen VJ (2002) High angular resolution diffusion imaging reveals intravoxel white matter fiber heterogeneity. Magn Reson Med 48:577–582. 10.1002/mrm.10268 12353272 10.1002/mrm.10268

[21] Tournier JD, Calamante F, Connelly A (2012) MRtrix: diffusion tractography in crossing fiber regions. Int J Imag Syst Tech 22:53–66. 10.1002/ima.22005

[22] Jeurissen B, Tournier JD, Dhollander T, Connelly A, Sijbers J (2014) Multi-tissue constrained spherical deconvolution for improved analysis of multi-shell diffusion MRI data. Neuroimage 103:411–426. 10.1016/j.neuroimage.2014.07.061 25109526 10.1016/j.neuroimage.2014.07.061

[23] Radwan AM, Sunaert S, Schilling K et al (2022) An atlas of white matter anatomy, its variability, and reproducibility based on constrained spherical deconvolution of diffusion MRI. Neuroimage 254:119029. 10.1016/j.neuroimage.2022.119029 35231632 10.1016/j.neuroimage.2022.119029 PMC10265547

[24] Zhang H, Schneider T, Wheeler-Kingshott CA, Alexander DC (2012) NODDI: practical in vivo neurite orientation dispersion and density imaging of the human brain. Neuroimage 61:1000–1016. 10.1016/j.neuroimage.2012.03.072 22484410 10.1016/j.neuroimage.2012.03.072

[25] Alves GS, Ferrer Carvalho A, de Amorim de Carvalho L et al (2017) Neuroimaging findings related to behavioral disturbances in Alzheimer’s disease: a systematic review. Curr Alzheimer Res 14:61–75. 10.2174/1567205013666160603010203 27298146 10.2174/1567205013666160603010203

[26] Zhou Y, Wei L, Gao S, Wang J, Hu Z (2023) Characterization of diffusion magnetic resonance imaging revealing relationships between white matter disconnection and behavioral disturbances in mild cognitive impairment: a systematic review. Front Neurosci 17:1209378. 10.3389/fnins.2023.1209378 37360170 10.3389/fnins.2023.1209378 PMC10285107

[27] Choo IH, Lee DY, Oh JS et al (2010) Posterior cingulate cortex atrophy and regional cingulum disruption in mild cognitive impairment and Alzheimer’s disease. Neurobiol Aging 31:772–779. 10.1016/j.neurobiolaging.2008.06.015 18687503 10.1016/j.neurobiolaging.2008.06.015

[28] Nakata Y, Sato N, Nemoto K et al (2009) Diffusion abnormality in the posterior cingulum and hippocampal volume: correlation with disease progression in Alzheimer’s disease. Magn Reson Imaging 27:347–354. 10.1016/j.mri.2008.07.013 18771871 10.1016/j.mri.2008.07.013

[29] Kantarci K, Murray EM, Schwarz CG et al (2017) White-matter integrity on DTI and the pathologic staging of Alzheimer’s disease. Neurobiol Aging 56:172–179. 10.1016/j.neurobiolaging.2017.04.024 28552181 10.1016/j.neurobiolaging.2017.04.024 PMC5523458

[30] Bergamino M, Walsh RR, Stokes AM (2021) Free-water diffusion tensor imaging improves the accuracy and sensitivity of white matter analysis in Alzheimer’s disease. Sci Rep 11:6990. 10.1038/s41598-021-86505-7 33772083 10.1038/s41598-021-86505-7 PMC7998032

[31] Bergamino M, Schiavi S, Daducci A, Walsh RR, Stokes AM (2022) Analysis of brain structural connectivity networks and white matter integrity in patients with mild cognitive impairment. Front Aging Neurosci 14:793991. 10.3389/fnagi.2022.793991 35173605 10.3389/fnagi.2022.793991 PMC8842680

[32] Folstein MF, Folstein SE, McHugh PR (1975) “Mini-mental state”. A practical method for grading the cognitive state of patients for the clinician.”. J Psychiatr Res 12:189–198. 10.1016/0022-3956(75)90026-6 1202204 10.1016/0022-3956(75)90026-6

[33] Nasreddine ZS, Phillips NA, Bédirian V et al (2005) The Montreal cognitive assessment, MoCA: a brief screening tool for mild cognitive impairment. J Am Geriatr Soc 53:695–699. 10.1111/j.1532-5415.2005.53221.x 15817019 10.1111/j.1532-5415.2005.53221.x

[34] FMRIB Software Library, Release 6.0 (2018). The University of Oxford. https://fsl.fmrib.ox.ac.uk/fsl/docs/#/. Accessed 9 Sept 2023

[35] MRtrix3 (2023) https://www.mrtrix.org/. Accessed 20 Sep 2023

[36] Tournier JD, Smith R, Raffelt D et al (2019) MRtrix3: A fast, flexible and open software framework for medical image processing and visualization. Neuroimage 202:116137. 10.1016/j.neuroimage.2019.116137 31473352 10.1016/j.neuroimage.2019.116137

[37] Schilling KG, Blaber J, Huo Y et al (2019) Synthesized b0 for diffusion distortion correction (Synb0-DisCo). Magn Reson Imaging 64:62–70 10.1016/j.mri.2019.05.008 PMC6834894 31075422

[38] Schilling KG, Blaber J, Hansen C et al (2020) Distortion correction of diffusion weighted MRI without reverse phase-encoding scans or field-maps. PLoS One 15:e0236418. 10.1371/journal.pone.0236418 32735601 10.1371/journal.pone.0236418 PMC7394453

[39] Garyfallidis E, Brett M, Amirbekian B et al (2014) Dipy, a library for the analysis of diffusion MRI data. Front Neuroinform 8:8. 10.3389/fninf.2014.00008 24600385 10.3389/fninf.2014.00008 PMC3931231

[40] Tournier JD, Calamante F, Connelly A (2010) Improved probabilistic streamlines tractography by 2nd order integration over fibre orientation distributions. In: Proceedings of the International Society for Magnetic Resonance in Medicine, 1670. John Wiley & Sons, Inc, New Jersey, NJ

[41] Fiscone C, Rundo L, Lugaresi A (2023) Assessing robustness of quantitative susceptibility-based MRI radiomic features in patients with multiple sclerosis. Sci Rep. 13:16239. 10.1038/s41598-023-42914-4 37758804 10.1038/s41598-023-42914-4 PMC10533494

[42] Benjamini Y, Hochberg Y (1995) Controlling the false discovery rate: a practical and powerful approach to multiple testing. J R Stat Soc B 57:289–300. 10.1111/j.2517-6161.1995.tb02031.x

[43] Heilbronner SR, Haber SN (2014) Frontal cortical and subcortical projections provide a basis for segmenting the cingulum bundle: implications for neuroimaging and psychiatric disorders. J Neurosci 34:10041–10054. 10.1523/JNEUROSCI.5459-13.2014 25057206 10.1523/JNEUROSCI.5459-13.2014 PMC4107396

[44] Testa C, Evangelisti S, Popeo M et al (2017) The effect of diffusion gradient direction number on corticospinal tractography in the human brain: an along-tract analysis. MAGMA 30:265–280. 10.1007/s10334-016-0600-1 28000087 10.1007/s10334-016-0600-1

[45] Casey KL, Minoshima S, Berger KL, Koeppe RA, Morrow TJ, Frey KA (1994) Positron emission tomographic analysis of cerebral structures activated specifically by repetitive noxious heat stimuli. J Neurophysiol 71:802–807. 10.1152/jn.1994.71.2.802 8176441 10.1152/jn.1994.71.2.802

[46] Tuladhar AM, van Norden AGW, de Laat KF et al (2015) White matter integrity in small vessel disease is related to cognition. Neuroimage Clin 7:518–524. 10.1016/j.nicl.2015.02.003 25737960 10.1016/j.nicl.2015.02.003 PMC4338206

[47] Skandalakis GP, Komaitis S, Kalyvas A et al (2020) Dissecting the default mode network: direct structural evidence on the morphology and axonal connectivity of the fifth component of the cingulum bundle. J Neurosurg 134:1334–1345. 10.3171/2020.2.JNS193177 32330886 10.3171/2020.2.JNS193177

[48] Metzler-Baddeley C, Jones DK, Steventon J, Westacott L, Aggleton JP, O’Sullivan MJ (2012) Cingulum microstructure predicts cognitive control in older age and mild cognitive impairment. J Neurosci 32:17612–17619. 10.1523/JNEUROSCI.3299-12.2012 23223284 10.1523/JNEUROSCI.3299-12.2012 PMC6621654

[49] Yamamoto M, Kushima I, Kimura H et al (2015) White matter microstructure between the pre-SMA and the cingulum bundle is related to response conflict in healthy subjects. Brain Behav 5:e00375. 10.1002/brb3.375 26516610 10.1002/brb3.375 PMC4614048

[50] Fox MD, Snyder AZ, Vincent JL, Corbetta M, Van Essen DC, Raichle ME (2005) The human brain is intrinsically organized into dynamic, anticorrelated functional networks. Proc Natl Acad Sci USA 102:9673–9678. 10.1073/pnas.0504136102 15976020 10.1073/pnas.0504136102 PMC1157105

[51] Chrastil ER, Sherrill KR, Hasselmo ME, Stern CE (2015) There and back again: hippocampus and retrosplenial cortex track homing distance during human path integration. J Neurosci 35:15442–15452. 10.1523/JNEUROSCI.1209-15.2015 26586830 10.1523/JNEUROSCI.1209-15.2015 PMC6605486

[52] Halliday G (2017) Pathology and hippocampal atrophy in Alzheimer’s disease. Lancet Neurol 16:862–864. 10.1016/S1474-4422(17)30343-5 29029840 10.1016/S1474-4422(17)30343-5

[53] Mitolo M, Zoli M, Testa C et al (2022) Neuroplasticity mechanisms in frontal brain gliomas: a preliminary study. Front Neurol 13:867048. 10.3389/fneur.2022.867048 35720068 10.3389/fneur.2022.867048 PMC9204970

[54] Zoli M, Talozzi L, Martinoni M et al (2021) From neurosurgical planning to histopathological brain tumor characterization: potentialities of arcuate fasciculus along-tract diffusion tensor imaging tractography measures. Front Neurol 12:633209. 10.3389/fneur.2021.633209 33716935 10.3389/fneur.2021.633209 PMC7952864

