# Supplementary material for: Connectivity related to major brain functions in Alzheimer disease progression: microstructural properties of the cingulum bundle and its subdivision using diffusion-weighted MRI
Source: Eur Radiol Exp. 2025 Mar 19;9:32. doi: 10.1186/s41747-025-00570-5 (PMC11923340; doi:10.1186/s41747-025-00570-5)
Supplement: Supplementary file 1 — Additional file 1: Fig. S1. Three-dimensional rendering of the ROIs used to reconstruct the three subdivisions of the cingulum bundle superimposed in the Montreal Neurological Institute space represented as a surface. ROI 1 (violet) and ROI 2 (teal) were used for the reconstruction of the subgenual subdivision as seed and “include” mode, respectively; ROI 3 (red) and ROI 4 (green) were used for the reconstruction of the retrosplenial subdivision as seed and “include” mode, respectively; ROI 5 (blue), ROI 6 (pink for the left hemisphere and yellow for the right hemisphere), and ROI 4 were used to reconstruct the parahippocampal subdivision as seed, “exclude”, and “include” mode, respectively. ROI Region of interest. Fig. S2. Orthogonal view of the thresholded (at 1%) overlap of the subdivisions of the cingulum bundle for the five middle-aged cognitive normal case (average representation): subgenual in cyan-blue color map (a); retrosplenial in red-yellow color map (b); and parahippocampal in light-dark green color map (c). The intensity scales are percentages associated to the minimum and maximum of the subjects represented by the overlap of the tracts. Fig. S3. (a) Example of axial views of diffusion-weighted images (b = 0 s/mm2) before distortion correction; distortions are evident especially in the frontal part of the brain. (b) The same axial view after Synb0-DISCO application and distortions correction, showing the frontal part of the brain restored. (c) The difference between a and b showing at a first glance, regions were the Synb0-DISCO have mostly corrected the distortions. Table S1. Coordinates of the center of gravity of the six ROIs and the midsagittal ROI used to reconstruct the subgenual, retrosplenial, parahippocampal subdivision of the cingulum bundle are reported in standard Montreal Neurological Institute space for both hemispheres. Table S2. Three-way ANOVA results for four NODDI metrics: tissue volume fraction, β-fraction, neurite dispersion index, [file 41747_2025_570_MOESM1_ESM.pdf]

# Connectivity related to major brain functions in Alzheimer disease progression: microstructural properties of the cingulum bundle and its subdivision using diffusion-weighted MRI

## ELECTRONIC SUPPLEMENTARY MATERIAL

### Methods

### MRI acquisition

DTI data were acquired with a multi-shell HARDI diffusion-weighted protocol at 3T (SIEMENS Magnetom Prisma Fit Scanner) using a 2D single-shot echo planar imaging (EPI) sequence with the following parameters: repetition time / echo time (TR/TE) = 3,400/71 ms; flip angle = 90°; acquisition matrix = 256 × 256; 2-mm isotropic voxel, no slice gap; 81 continuous axial oblique slices; number of excitations = 1, acceleration factor = 2; total acquisition time 7:10 min:s. The diffusion-weighting schedule followed this design: 114 diffusion-encoding directions with $b$ = 0 s/mm$^2$ ($n$ = 13), $b$ = 500 s/mm$^2$ ($n$ = 6), $b$ = 1,000 s/mm$^2$ ($n$ = 48), and $b$ = 2000 s/mm$^2$ ($n$ = 60), all with a posterior–anterior (PA) phase-encoding direction. The DTI acquisition protocol did not include an inverted phase encoding direction acquisition for any diffusion weighting factors, thus not allowing the use of the information from the two phase-encoding directions to correct echo-planar imaging (EPI) distortion artefacts in the EPI volumes.

Accelerated sagittal T1-weighted anatomical images were acquired using a 3D magnetization prepared rapid acquisition gradient echo (MPRAGE) sequence: 208 continuous sagittal slices with 1-mm isotropic voxel; no slice gap; TE = 2.95 ms, TR = 2300 ms, inversion time (IT) = 900 ms; flip angle = 9°; acquisition matrix = 208 × 208; in-plane acceleration factor = 2.

### DWI postprocessing

Diffusion-weighted images were skull-stripped using the FSL bet tool [1s]. Image denoising was performed with the MRtrix3 *dwidenoise* function [2s] using a principal component analysis approach.

A recent tool, Synb0-DISCO, based on the use of deep learning to synthesize an undistorted non-diffusion-weighted image from the structural image was applied for distortions correction

This tool performs susceptibility distortion correction when the DTI protocol acquisition does not include specific image acquisition sequences for distortion correction such as the reverse phase-encoded scans. Briefly, an "undistorted" b=0 image that matches the geometry of structural $T_{1w}$ images is synthesized, matching the contrast from diffusion images. The 'undistorted' image can successively be used in standard workflows as in the following. The use of Synb0-DISCO has

been mandatory since the DW-EP images, both at b=0 s/mm$^2$ and at b > 0 s/mm$^2$, are greatly distorted.

Subsequently, the output of Synb0-DISCO was used for the FSL *topup* function [3s, 4s], and then, a combined correction for susceptibility, eddycurrent effects, and signal dropout, most commonly induced by subject movement, was performed by FSL *eddy* [5s], based on the *topup* estimates.

The DWI data obtained by a multi-shell HARDI protocol were modeled at the voxel level using functions of the MRtrix3 toolkit. In particular, the response function for spherical deconvolution was modeled using the Dhollander algorithm within *dwi2fod* [6s, 7s]. Fiber orientation distributions were estimated using multi-shell multi-tissue spherical deconvolution (*msmt_csd*) to infer three parameters from three tissue compartments, two with isotropic diffusion characteristics, one representing restricted diffusion and labeled ”grey matter” (GM) and the other representing unhindered diffusion and labeled cerebrospinal fluid (CSF); finally one component with anisotropic characteristics modeled as a tensor, was labeled ”white matter” (WM) [8s]. In the present, we focused only on the WM component.

Further characterization of the diffusion properties of tissue within each voxel was provided by FSL *dtifit* based on the diffusion tensor, which was used to estimate voxel fractional anisotropy (FA), mean diffusivity (MD), and radial diffusivity (RD).

The Diffusion Microstructure Imaging in Python (Dmipy) package [9s] was used to process data with the Bingham-NODDI model. Tissue volume fraction, b-fraction, Neurite Dispersion Index (NDI) and Orientation Dispersion Index (ODI) were calculated.

**Image registration**

The alignment of images in the diffusion space to the $T_1$ space and vice versa have been performed by FSL registration tools ( https://fsl.fmrib.ox.ac.uk/fsl/fslwiki ); specifically, *flirt* [10s, 11s] for linear registration and *fnirt* for non-linear registration [12s]. Additionally, the standard MNI (ICBM-152) space available in FSL was used to register intermediate results in a common space.

**Cingulum bundle subdivisions reconstruction**

To reconstruct the three subdivisions, ROIs in the diffusion images space were used, with dimensions of 12 × 12 × 2 mm$^3$, except for the midsagittal ROI with 4 mm in the *z* direction. For each tract, three types of ROIs were identified: the “seed” ROI, from which the integration of the streamline pathway starts; the “include” ROI, which selects only the streamlines passing through this region; finally, one or more "exclude" ROIs, which eliminates all the streamlines that go through these areas. A total of 6 ROIs (labelled respectively 1, 2, 3, 4, 5, 6) per hemisphere were used, and the mid-sagittal ROI. The SurfIce software (https://www.nitrc.org/plugins/mwiki/index.php/surfice:MainPage) was used for the projection of

voxel-wise data onto a surface mesh and to display the ROIs in three dimensions aligned onto the Montreal Neurological Institute (MNI) standard (Supplemental Fig. S1, Supplementary Table S1).

The ROIs were first identified in the diffusion spaces of the 5 middle-aged CN subjects, as previously mentioned. For each subject, the ROIs were then registered to the MNI space. For each one, the final MNI-ROI was identified by retaining only those portions where there was an overlap in at least 3 out of the 5 subjects. This procedure was carried out to define ROIs that considered the variability across subjects. The result is shown in Fig. S1, as the six ROIs for each hemisphere, aligned onto the MNI space. Once the necessary ROIs for constructing the three subdivisions were identified in the MNI space, they were registered back to the diffusion space of each single subject thus allowing to automatically have a set of ROIs to reconstruct the three subdivisions of the CB.

The three subdivisions were reconstructed again in the diffusion space for each of the 5 subjects. This step served two purposes. The first purpose was to define a mask for the CN, MCI and AD group (the probabilistic approach can be affected by false positive streamlines and the definition of a mask in the MNI space allows for a common delineation of the bundle boundaries and a truncation of the streamlines when they exceed target zones). The second one was to define these ROIs in a template space to use them to reconstruct the bundles in new subjects by automatically back-registering them on the diffusion space of the new subject.

The three subdivisions for each of the 5 middle-aged CNs were constructed using the MNI-ROIs appropriately registered in the diffusion space; the bundles were normalized with respect to their maximum value and registered in the MNI space; for each subdivision, the 5 results were overlapped, and a binary mask was defined by applying a threshold of 1% to the sum of these overlaps (Fig. S2).

*Subgenual subdivision.* To reconstruct the subgenual component of the CB the first ROI 1 (violet in Fig. S1) was placed in the DW images, on the third coronal slice caudal to the most anterior part of the genu. It is located 4mm inside the corpus callosum. This ROI was used as the seed region to initiate the propagation of the streamlines. To position the second ROI 2 (teal in Fig. S1), the rostral-caudal midpoint of the body of the corpus callosum was identified. A plane was collocated as the midpoint between the back of the curve of the genu (the most posterior part where the curvature is the greatest) and the front of the splenium (its most anterior part at the flexure). From this point, the ROI was moved caudally by 10mm. This ROI was used as an “include” ROI. Finally, the midsagittal ROI was used as the exclusion parameter.

*Retrosplenial subdivision.* The ROI 3 (red in Fig. S1) has been positioned by moving 10 mm rostrally from the mid-way point of the plane previously identified. One side touches the midsagittal plane, and axially it is located 2 mm superior to ROI 2. This ROI was used as the seed for the retrosplenial subdivision. The location of the ROI 4 has been positioned in an axial plane (green in Fig. S1). It was identified by finding the most ventral plane of the splenium and selecting the horizontal section 6 mm above the base of the splenium axially, and 4 mm inside the splenium

coronally. This ROI was used as an “include” ROI. Finally, the midsagittal plane was used as the exclusion ROI.

*Parahippocampal subdivision.* The ROI 5 used as a seed region (blue in Fig. S1) was located on the axial plane, 4 mm inferior and 6 mm anterior to ROI 4. ROI 4 was used as an “include” ROI (green in Fig. S1), while ROI 3 was used as an “exclude” ROI. The other two exclusion ROIs are the midsagittal ROI and ROI 6 (pink for the left hemisphere and yellow for the right hemisphere in Fig. S1). ROI 6 was carefully drawn for each subject to exclude streamlines that curved in a semi-circle shape around the corpus callosum, following a path in the coronal axis rather than the sagittal axis. It has been positioned on a sagittal plane. Starting from the caudal edge of ROI 4, it has been located approximately 6 mm lateral to this ROI, just above the lateral ventricles. The size of this ROI is 14mm x 14mm x 2mm.

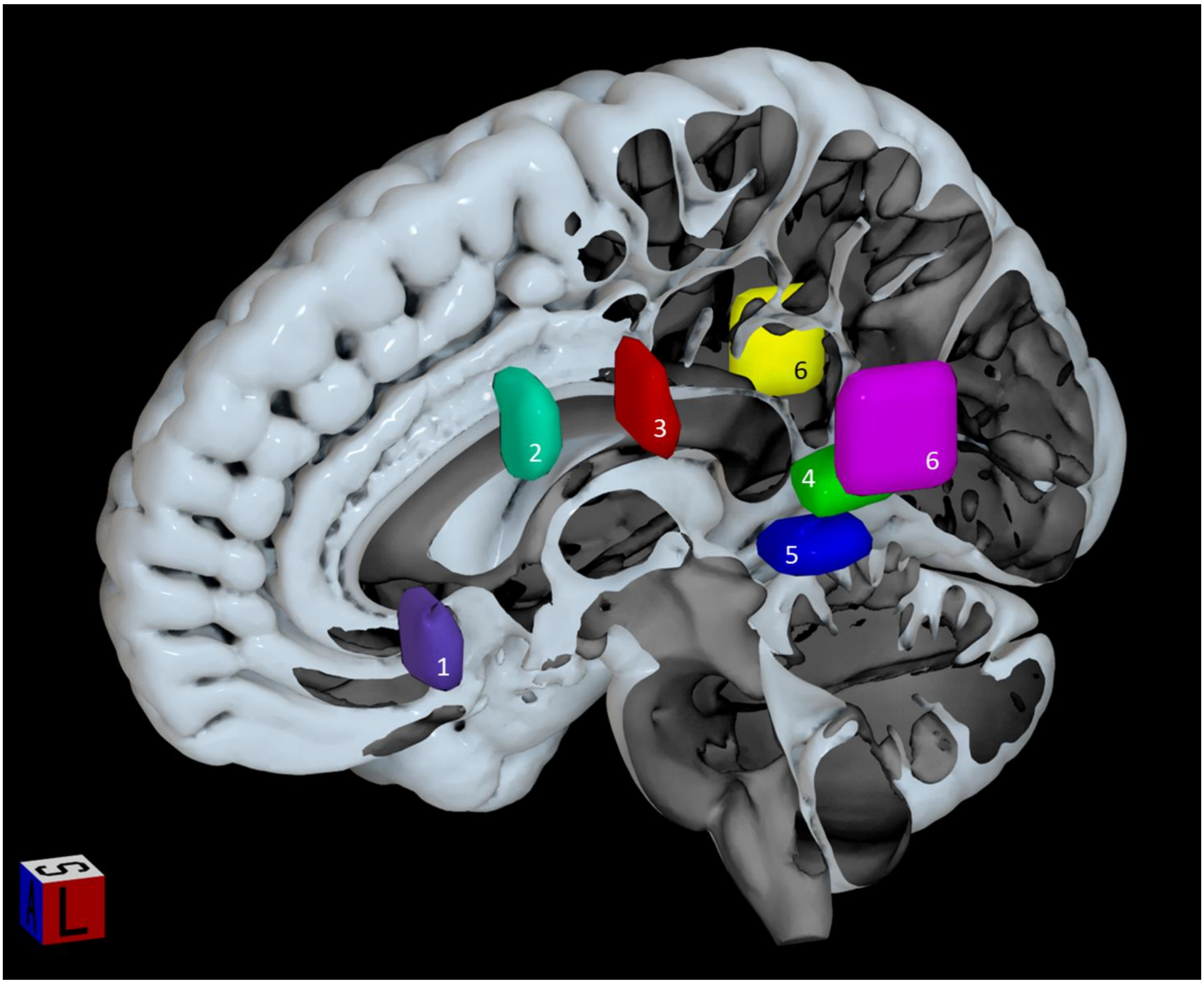


**Fig. S1.** Three-dimensional rendering of the ROIs used to reconstruct the three subdivisions of the cingulum bundle superimposed in the Montreal Neurological Institute space represented as a surface. ROI 1 (violet) and ROI 2 (teal) were used for the reconstruction of the subgenual subdivision as seed and “include” mode, respectively; ROI 3 (red) and ROI 4 (green) were used for the reconstruction of the retrosplenial subdivision as seed and “include” mode, respectively; ROI 5 (blue), ROI 6 (pink for the left hemisphere and yellow for the right hemisphere), and ROI 4 were used to reconstruct the parahippocampal subdivision as seed, “exclude”, and “include” mode, respectively. *ROI* Region of interest.

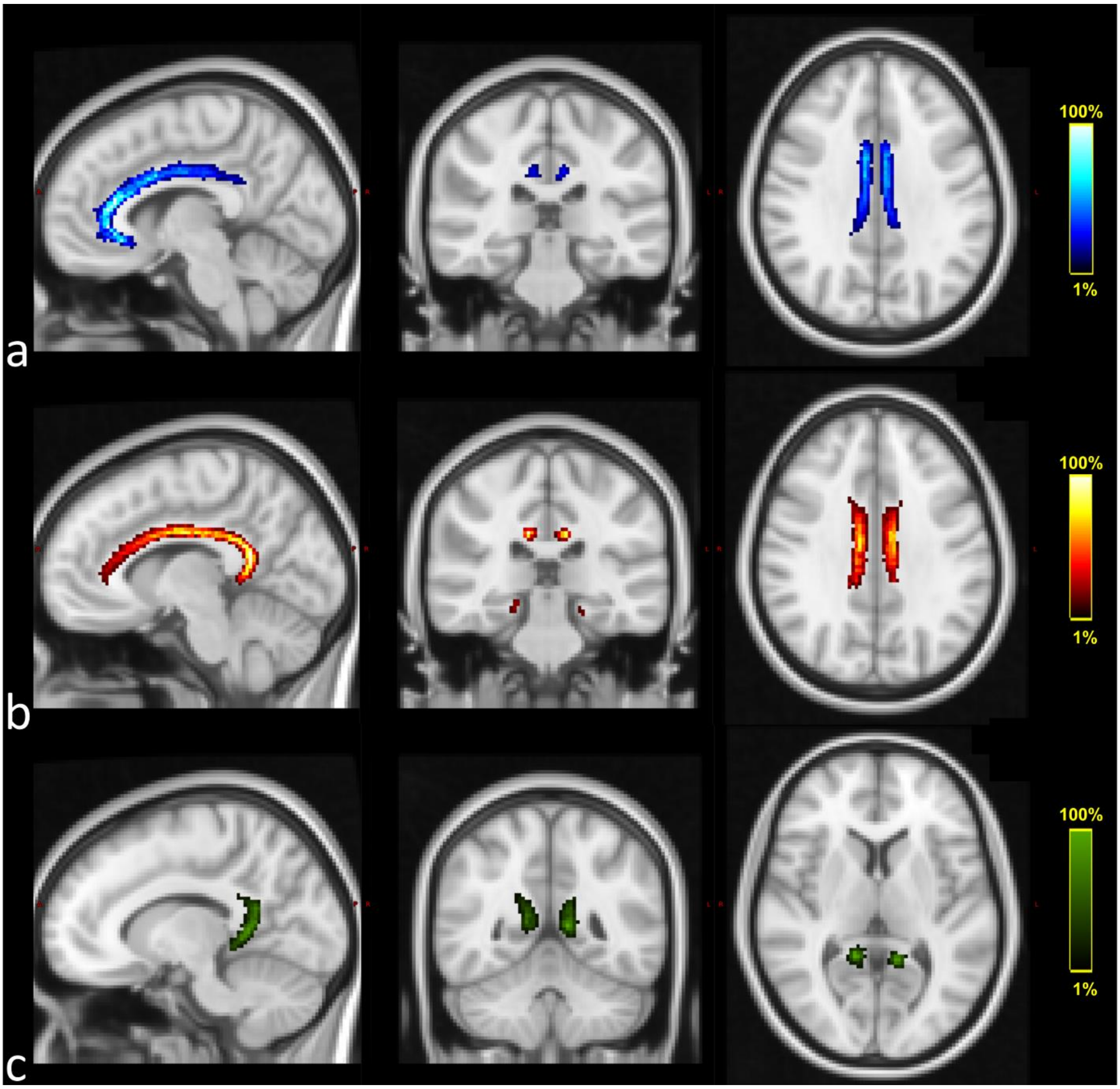


**Fig. S2.** Orthogonal view of the thresholded (at 1%) overlap of the subdivisions of the cingulum bundle for the five middle-aged cognitive normal case (average representation): subgenual in cyan-blue color map (**a**); retrosplenial in red-yellow color map (**b**); and parahippocampal in light-dark green color map (**c**). The intensity scales are percentages associated to the minimum and maximum of the subjects represented by the overlap of the tracts.

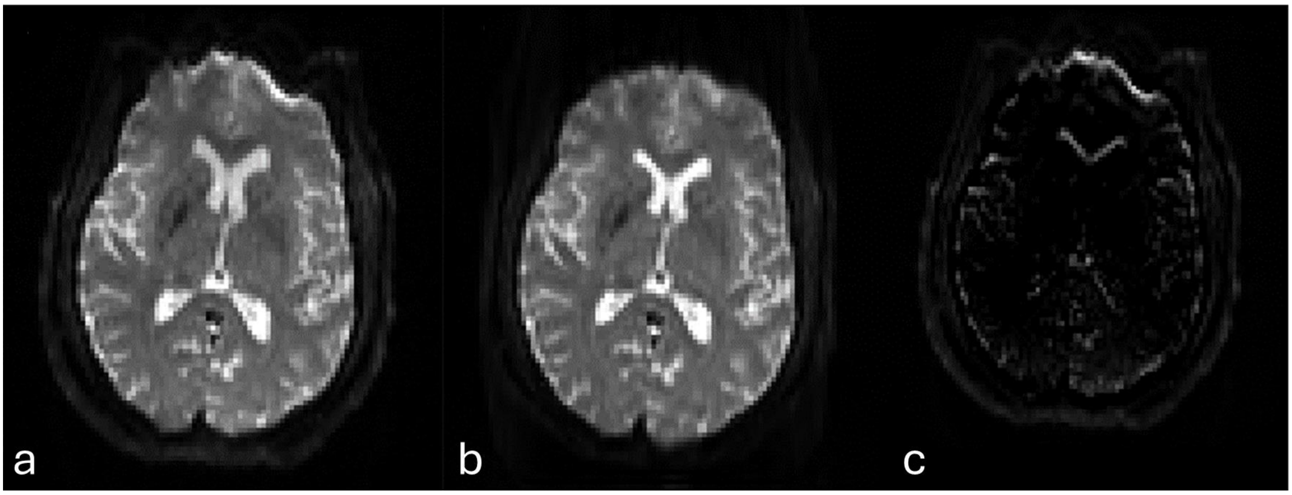


**Fig. S3.** (**a**) Example of axial views of diffusion-weighted images ($b$ = 0 s/mm$^2$) before distortion correction; distortions are evident especially in the frontal part of the brain. (**b**) The same axial view after Synb0-DISCO application and distortions correction, showing the frontal part of the brain restored. (**c**) The difference between **a** and **b** showing at a first glance, regions were the Synb0-DISCO have mostly corrected the distortions.

**Table S1.** Coordinates of the center of gravity of the six ROIs and the midsagittal ROI used to reconstruct the subgenual, retrosplenial, parahippocampal subdivision of the cingulum bundle are reported in standard Montreal Neurological Institute space for both hemispheres

| | *x* (mm) | *y* (mm) | *z* (mm) |
|---|---|---|---|
| 1_left | -7.94 | 24.02 | -6.68 |
| 1_right | 8.90 | 24.22 | -6.88 |
| 2_left | -7.28 | 6.57 | 28.58 |
| 2_right | 8.41 | 7.01 | 28.04 |
| 3_left | -8.03 | -13.65 | 30.96 |
| 3_right | 9.71 | -13.45 | 31.27 |
| 4_left | -8.01 | -48.44 | 12.92 |
| 4_right | 9.57 | -48.47 | 12.81 |
| 5_left | -8.60 | -43.27 | 2.40 |
| 5_right | 10.17 | -42.80 | 2.70 |
| 6_left | -22.00 | -52.72 | 25.72 |
| 6_right | 26.09 | -49.30 | 24.80 |
| Midsagittal | 1.00 | -18.00 | 18.00 |

The role of each ROI in the process of reconstruction is reported in the main manuscript. *ROI* region of interest.

**Table S2.** Three-way ANOVA results for four NODDI metrics: tissue volume fraction, β-fraction, neurite dispersion index, and orientation dispersion index

| Tissue volume fraction | | | β-fraction | | | Neurite dispersion index | | | Orientation dispersion index | | |
|---|---|---|---|---|---|---|---|---|---|---|---|
| Effect | F | *p*-value | Effect | F | *p*-value | Effect | F | *p*-value | Effect | F | *p*-value |
| Subdivision (SGC, RSC, PHC) | 187.01 | < **0.001** | Subdivision (SGC, RSC, PHC) | 75.80 | < **0.001** | Subdivision (SGC, RSC, PHC) | 39.74 | < **0.001** | Subdivision (SGC, RSC, PHC) | 74.15 | < **0.001** |
| Side (Left, Right) | < 0.001 | 0.980 | Side (Left, Right) | 8.46 | **0.004** | Side (Left, Right) | 0.003 | 0.956 | Side (Left, Right) | 2.75 | 0.098 |
| Group (AD, MCI, CN) | 3.72 | **0.026** | Group (AD, MCI, CN) | 1.41 | 0.246 | Group (AD, MCI, CN) | 25.86 | < **0.001** | Group (AD, MCI, CN) | 0.70 | 0.499 |
| Subdivision:Group | 1.60 | 0.176 | Subdivision:Group | 0.64 | 0.633 | Subdivision:Group | 0.06 | 0.994 | Subdivision:Group | 0.35 | 0.842 |
| Subdivision:Side | 1.32 | 0.270 | Subdivision:Side | 4.61 | **0.011** | Subdivision:Side | 0.89 | 0.412 | Subdivision:Side | 4.41 | **0.013** |
| Side:Group | 0.43 | 0.649 | Side:Group | 0.15 | 0.859 | Side:Group | 0.20 | 0.819 | Side:Group | 1.09 | 0.339 |
| Subdivision:Tract:Side | 0.03 | 0.998 | Subdivision:Tract:Side | 1.47 | 0.213 | Subdivision:Tract:Side | 0.02 | 0.999 | Subdivision:Tract:Side | 0.69 | 0.600 |

In bold the significant adjusted *p*-value (< 0.05). *AD* Alzheimer disease, *CN* Cognitively normal, *MCI* Mild cognitive impairment, *PHC* Parahippocampal cingulum, *RSC* Retrosplenial cingulum, *SGC* Subgenual cingulum.

**Table S3.** Post hoc analyses for group differences performed by the Tukey's HSD test applying the Benjamini- Hochberg correction for the adjusted *p*-values.

| Tissue volume fraction | | | | Neurite dispersion index | | | |
|---|---|---|---|---|---|---|---|
| Group | Subdivision | Adjusted *p*-value | Effect size | Group | Subdivision | Adjusted *p*-value | Effect size |
| AD < CN | SGC | 0.984 | 0.07 | AD < CN | SGC | **0.026** | 1.01 |
| AD > MCI | SGC | 0.947 | 0.11 | AD < MCI | SGC | 0.781 | 0.28 |
| MCI < CN | SGC | 0.843 | 0.22 | MCI < CN | SGC | 0.053 | 0.84 |
| AD < CN | RSC | 0.787 | 0.34 | AD < CN | RSC | **0.034** | 0.99 |
| AD > MCI | RSC | 0.948 | 0.10 | AD < MCI | RSC | 0.856 | 0.22 |
| MCI < CN | RSC | 0.500 | 0.40 | MCI < CN | RSC | **0.049** | 0.84 |
| AD < CN | PHC | 0.321 | 0.75 | AD < CN | PHC | **0.005** | 1.08 |
| AD < MCI | PHC | 0.996 | 0.03 | AD < MCI | PHC | 0.578 | 0.15 |
| MCI < CN | PHC | 0.253 | 0.56 | MCI < CN | PHC | **0.019** | 0.97 |

In bold the significant adjusted *p*-values (< 0.05). Effect size computed as "*Cohen's d*" (small = 0.2; medium = 0.5; large = 0.8). *AD* Alzheimer disease, *CN* Cognitively normal, MCI Mild Cognitive Impairment, *PHC* Parahippocampal cingulum, *RSC* Retrosplenial cingulum, *SGC* Subgenual cingulum.

**Table S4.** Post hoc analyses for subdivision differences performed by the Tukey's HSD test applying the Benjamini- Hochberg correction for the adjusted *p*-values

| Tissue volume fraction | | | β-fraction | | | Neurite dispersion index | | | Orientation dispersion index | | |
|---|---|---|---|---|---|---|---|---|---|---|---|
| Subdivision | Group | Adjusted *p*-value | Subdivision | Group | Adjusted *p*-value | Subdivision | Group | Adjusted *p*-value | Subdivision | Group | Adjusted *p*-value |
| RSC > PHC | CN | **< 0.001** | RSC > PHC | CN | **< 0.001** | RSC > PHC | CN | **0.002** | RSC < P | CN | **<0 .001** |
| SGC > PHC | CN | **< 0.001** | SGC > PHC | CN | **< 0.001** | SGC > PHC | CN | 0.059 | S < P | CN | **0.011** |
| RSC < SGC | CN | **< 0.001** | RSC < SGC | CN | 0.188 | RSC < SGC | CN | 0.426 | RSC < S | CN | **0.002** |
| RSC > PHC | AD | **< 0.001** | RSC > PHC | AD | **0.004** | RSC > PHC | AD | **0.005** | RSC < P | AD | **< 0.001** |
| SGC > PHC | AD | **< 0.001** | SGC > PHC | AD | **0.004** | SGC > PHC | AD | **0.041** | S < P | AD | **0.444** |
| RSC < SGC | AD | **0.031** | RSC < SGC | AD | 0.992 | RSC < SGC | AD | 0.631 | RSC < S | AD | **0.017** |
| RSC > PHC | MCI | **< 0.001** | RSC > PHC | MCI | **< 0.001** | RSC > PHC | MCI | **< 0.001** | RSC < P | MCI | **< 0.001** |
| SGC > PHC | MCI | **< 0.001** | SGC > PHC | MCI | **< 0.001** | SGC > PHC | MCI | **0.005** | S < P | MCI | **0.007** |
| RSC < SGC | MCI | **0.004** | RSC < SGC | MCI | 0.125 | RSC < SGC | MCI | 0.457 | RSC < S | MCI | **< 0.001** |

In bold the significant adjusted *p*-values (< 0.05). *AD* Alzheimer disease, *CN* Cognitively normal, *MCI* Mild cognitive impairment, *PHC* Parahippocampal cingulum, *RSC* Retrosplenial cingulum, *SGC* Subgenual cingulum.

## Supplemental references


1s. Smith S (2002) Fast robust automated brain extraction. Hum Brain Mapp 17(3):143-155. https://doi.org/10.1002/hbm.10062

2s. Tournier JD, Smith R, Raffelt D et al (2019) MRtrix3: A fast, flexible and open software framework for medical image processing and visualization. NeuroImage 202:116137. https://doi.org/10.1016/j.neuroimage.2019.116137

3s. Andersson J, Skare S, Ashburner J (2003) How to correct susceptibility distortions in spin-echo echo planar images: application to diffusion tensor imaging. Neuroimage 20(2):870-888. https://doi.org/10.1016/S1053-8119(03)00336-7

4s. Smith S, Jenkinson M, Woolrich M et al (2004) Advances in functional and structural MR image analysis and implementation as FSL. NeuroImage 23(S1):208-219. https://doi.org/10.1016/j.neuroimage.2004.07.051

5s. Andersson JLR, Sotiropoulos SN (2016) An integrated approach to correction for off-resonance effects and subject movement in diffusion MR imaging. NeuroImage 125:1063–1078. https://doi.org/10.1016/j.neuroimage.2015.10.019

6s. Dhollander T, Raffelt D, Connelly A (2016) Unsupervised 3-tissue response function estimation from single-shell or multi-shell diffusion MR data without a co-registered T1 image. ISMRM Workshop on Breaking the Barriers of Diffusion MRIAt: Lisbon, Portugal, pp. 5.

7s. Dhollander T, Mito R, Raffelt D, Connelly A (2019) Improved white matter response function estimation for 3-tissue constrained spherical deconvolution. Proceedings of the ISMRM27th AnnualMeeting & Exhibition. Montréal, QC, Canada.

8s. Jeurissen B, Tournier JD, Dhollander T, Connelly A, Sijbers J (2014) Multi-tissue constrained spherical deconvolution for improved analysis of multi-shell diffusion MRI data. NeuroImage 103:411–426. https://doi.org/10.1016/j.neuroimage.2014.07.061

9s. Fick RHJ, Wassermann D, Deriche R (2019) The Dmipy Toolbox: diffusion MRI multi-compartment modeling and microstructure recovery made easy. Front Neuroinform 13:64. https://doi.org/10.1016/j.neuroimage.2014.07.06110.3389/fninf.2019.00064

10s. Jenkinson M, Smith S (2001) A global optimisation method for robust affine registration of brain images. Med Image Anal 5(2):143-156. https://doi.org/10.1016/S1361-8415(01)00036-6

11s. Jenkinson M, Bannister P, Brady J, Smith S (2002) Improved optimisation for the robust and accurate linear registration and motion correction of brain images. NeuroImage 17(2):825-841. https://doi.org/10.1006/nimg.2002.1132

12s. Andersson J, Jenkinson M, Smith S (2007) Non-linear registration, aka spatial normalisation. FMRIB technical report TR07JA2. FMRIB Analysis Group of the University of Oxford 2(1):1-22.
